# Supplementary material for: Challenges in clinical diagnosis of Clade I Mpox: Highlighting the need for enhanced diagnostic approaches
Source: PLoS Negl Trop Dis. 2024 Jun 24;18(6):e0012087. doi: 10.1371/journal.pntd.0012087 (PMC11226010; doi:10.1371/journal.pntd.0012087)
Supplement: S1 Appendix — (PDF) [file pntd.0012087.s001.pdf]

Questionnaire: Inter-rater agreement and reliability of Clade 1 mpox lesion assessment study

SCREEN 1

**Eligibility screening tool**
Please select ONE of the following options that best describes your clinical experience with mpox:<br>a. I am a clinician who treats patients with Clade I mpox virus disease and I have personally been involved in the care of at least 1 mpox patient in the last 1 year, or I am a clinician who is involved in mpox clinical research and seen patients with Clade I mpox in the last 1 year<br>b. I am not responsible for the clinical management of patients with mpox

SCREEN 2

**Participant information sheet and consent box**
[Insert participant information sheet text]<br><br>If you have read the information above and agree to participate with the understanding that the data you submit will be processed accordingly, please tick the box below to start.
[If consent box ticked = proceed to screen 3]

SCREEN 3

**Lesion definitions**
In this questionnaire we will be using the following lesion classifications and definitions, which are in line with the World Health Organisation (WHO) working definitions:<br><br>1. Active lesion: defined as any of the following –<br>a. Early lesions: Typically 1–3 mm that appear solid, are known as papules. Other lesions may develop a clear fluid-filled appearance known as a vesicle or, more often, will appear to contain white material (pustule/pseudo-pustule).<br>b. As lesions progress, central involution of the lesion may lead to a torus shape (umbilicated pseudo-pustule) that resembles a bagel or doughnut. A scale crust often develops first in the umbilicated central area of evolving skin lesions.<br>c. Erosions and ulcers may also occur, which are also considered active.Superficial loss of a portion of the epidermis is an erosion, whereas full-thickness loss of the entire epidermis is an ulcer.<br><br>2. Crusted and scabbed lesions: Evolving lesions may develop a surface of thick surface scale (stratum corneum), yellow serous crust, haemorrhagic crust or scab (eschar).Crusted lesions are considered infectious until the underlying lesion has completely resolved and the surface crust has been replaced by a full layer of epidermis.At this stage, most of the lesion should have crust without surrounding skin thickening (induration).

3. Resolved and healed lesions: Lesions are considered resolved once the lesion is no longer raised above the skin and any residual crust has resolved (desquamation).The underlying skin at the site of the lesion should appear intact.The skin may appear red or pink initially.Scar formation or “post-inflammatory” pigmentary changes (hyperpigmentation, hypopigmentation) may occur and persist for several weeks or longer after lesions have resolved.These “residual” changes are still considered a resolved/healed lesion.
Proceed to questionnaire

QUESTIONNAIRE

**Question 1**
In the last 1 year, approximately how many patients with confirmed mpox virus disease have you personally managed:<br>1. <5<br>2. 5-10<br>3. 10-20<br>4. 20-50<br>5. 50+

**Question 2**
On a scale of 1 to 10, how confident are you in assessing a mpox lesion?<br>Note: 1 = no confidence and 10 = complete confidence<br><br>[1-10 drop down list]

**Question 3**
In which country to do you usually work?<br><br>[insert dropdown list of countries]

Question 4

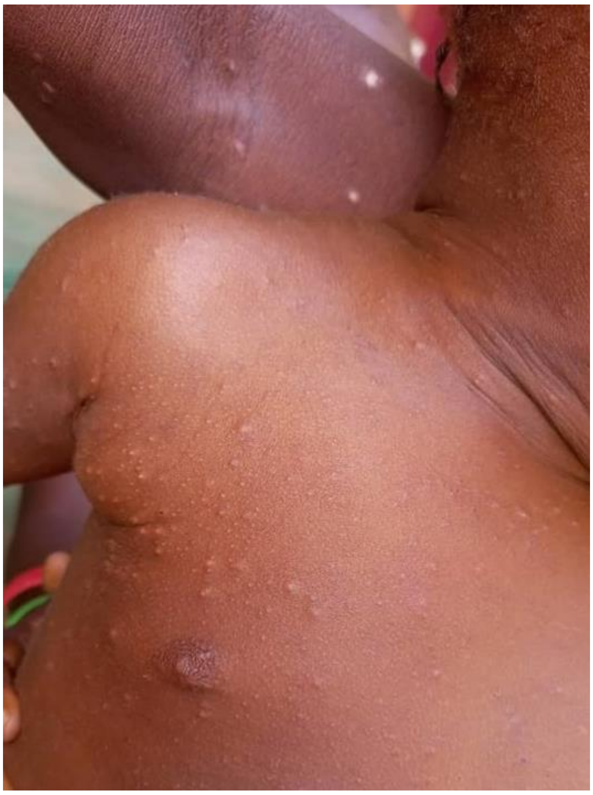

A. Based on the lesion presentation in this image, which of the following diagnoses is more likely? Select ONE
   1. Mpox
   2. Chickenpox (varicella)

B. What types of lesion do you see on both people? Select MULTIPLE
   1. Active lesion (papules, vesicles, pustule/pseudo-pustule, umbilicated pseudo-pustule, erosion, or ulcer)
   2. Crusted and scabbed lesion (thick surface scale (stratum corneum), yellow serous crust, haemorrhagic crust or scab (eschar)).
   3. Resolved and healed lesion (lesion is no longer raised above the skin and any residual crust has resolved (desquamation)).
   4. Unable to classify

Question 5

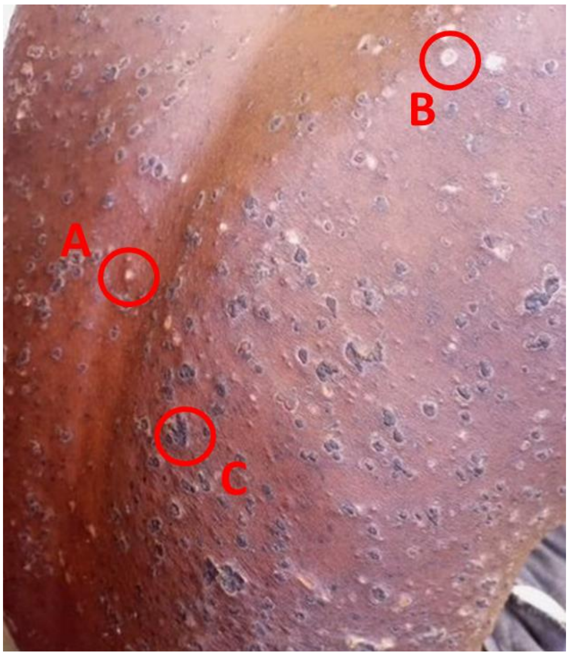

A. Based on the lesion presentation in this image, which of the following diagnoses is more likely? Select ONE
   1. Mpox
   2. Chickenpox (varicella)

B. Three different types of lesion are circled in the image. What type of lesion is lesion A? Select ONE
   1. Active lesion (papules, vesicles, pustule/pseudo-pustule, umbilicated pseudo-pustule, erosion, or ulcer)
   2. Crusted and scabbed lesion (thick surface scale (stratum corneum), yellow serous crust, haemorrhagic crust or scab (eschar)).
   3. Resolved and healed lesion (lesion is no longer raised above the skin and any residual crust has resolved (desquamation)).
   4. Unable to classify

C. What type of lesion is lesion B? Select ONE
   1. Active lesion (papules, vesicles, pustule/pseudo-pustule, umbilicated pseudo-pustule, erosion, or ulcer)
   2. Crusted and scabbed lesion (thick surface scale (stratum corneum), yellow serous crust, haemorrhagic crust or scab (eschar)).
   3. Resolved and healed lesion (lesion is no longer raised above the skin and any residual crust has resolved (desquamation)).
   4. Unable to classify

D. What type of lesion is lesion C? Select ONE
   5. Active lesion (papules, vesicles, pustule/pseudo-pustule, umbilicated pseudo-pustule, erosion, or ulcer)
   6. Crusted and scabbed lesion (thick surface scale (stratum corneum), yellow serous crust, haemorrhagic crust or scab (eschar)).
   7. Resolved and healed lesion (lesion is no longer raised above the skin and any residual crust has resolved (desquamation)).
   8. Unable to classify

Question 6

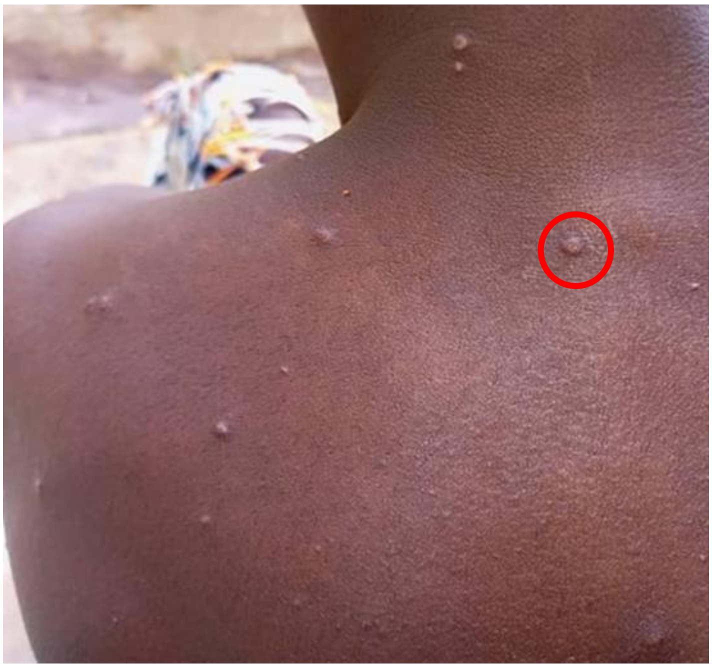

A. Based on the lesion presentation in this image, which of the following diagnoses is more likely? Select ONE
   1. Mpox
   2. Chickenpox (varicella)

B. What type of lesion do you see circled in red in this image? Select ONE
   1. Active lesion (papules, vesicles, pustule/pseudo-pustule, umbilicated pseudo-pustule, erosion, or ulcer)
   2. Crusted and scabbed lesion (thick surface scale (stratum corneum), yellow serous crust, haemorrhagic crust or scab (eschar)).
   3. Resolved and healed lesion (lesion is no longer raised above the skin and any residual crust has resolved (desquamation)).
   4. Unable to classify

Question 7

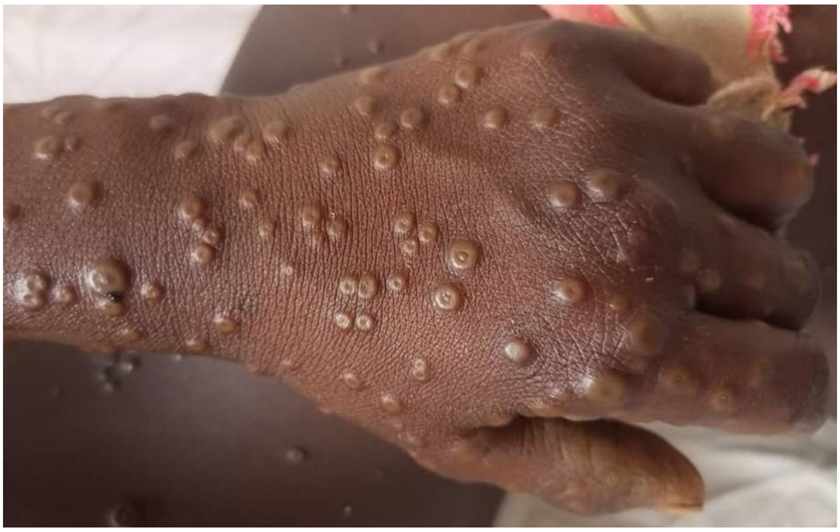

A. Based on the lesion presentation in this image, which of the following diagnoses is more likely? Select ONE
   1. Mpox
   2. Chickenpox (varicella)

B. What types of lesion do you see in this image? Select ONE
   1. Active lesion (papules, vesicles, pustule/pseudo-pustule, umbilicated pseudo-pustule, erosion, or ulcer)
   2. Crusted and scabbed lesion (thick surface scale (stratum corneum), yellow serous crust, haemorrhagic crust or scab (eschar)).
   3. Resolved and healed lesion (lesion is no longer raised above the skin and any residual crust has resolved (desquamation)).
   4. Unable to classify

Question 8

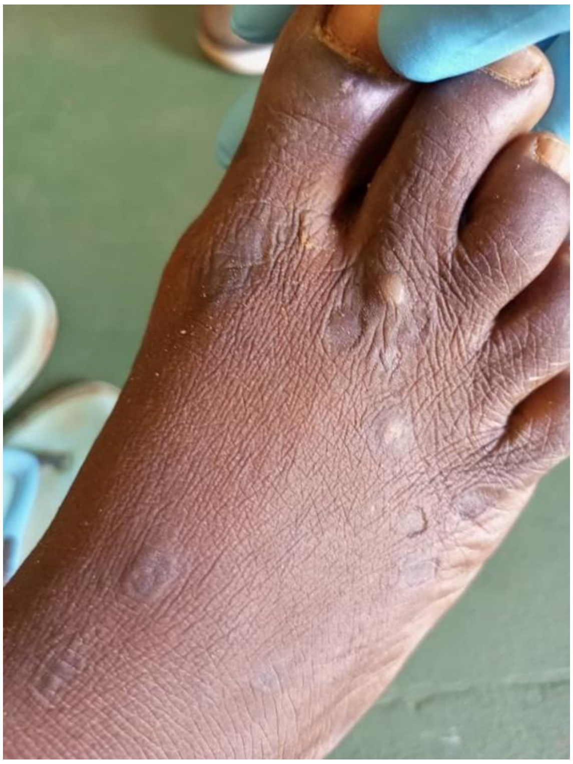

A. Based on the lesion presentation in this image, which of the following diagnoses is more likely? Select ONE
   1. Mpox
   2. Chickenpox (varicella)

B. What types of lesion do you see in this image? Select MULTIPLE
   1. Active lesion (papules, vesicles, pustule/pseudo-pustule, umbilicated pseudo-pustule, erosion, or ulcer)
   2. Crusted and scabbed lesion (thick surface scale (stratum corneum), yellow serous crust, haemorrhagic crust or scab (eschar)).
   3. Resolved and healed lesion (lesion is no longer raised above the skin and any residual crust has resolved (desquamation)).
   4. Unable to classify

Question 9

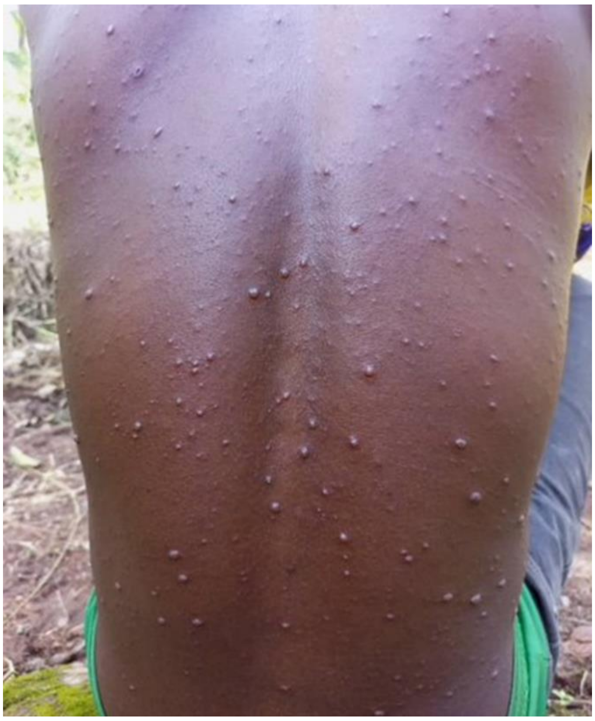

A. Based on the lesion presentation in this image, which of the following diagnoses is more likely? Select ONE
   1. Mpox
   2. Chickenpox (varicella)

B. What types of lesion do you see in this image? Select MULTIPLE
   1. Active lesion (papules, vesicles, pustule/pseudo-pustule, umbilicated pseudo-pustule, erosion, or ulcer)
   2. Crusted and scabbed lesion (thick surface scale (stratum corneum), yellow serous crust, haemorrhagic crust or scab (eschar)).
   3. Resolved and healed lesion (lesion is no longer raised above the skin and any residual crust has resolved (desquamation)).
   4. Unable to classify

Question 10

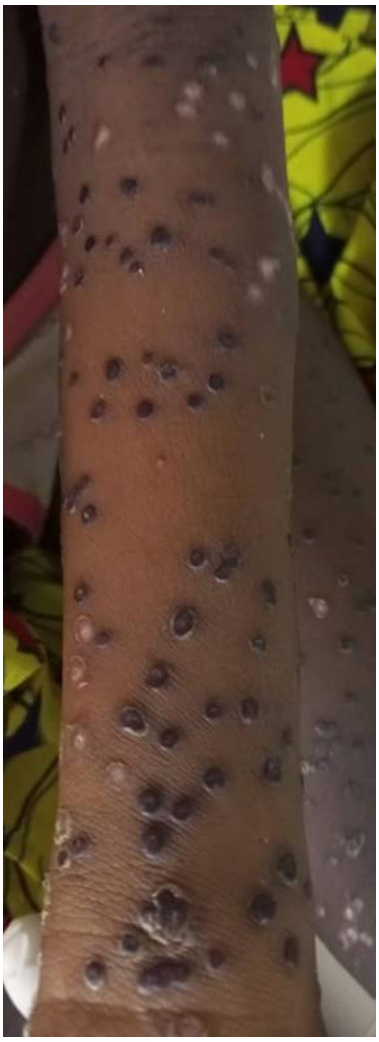

A. Based on the lesion presentation in this image, which of the following diagnoses is more likely? Select ONE
   1. Mpox
   2. Chickenpox (varicella)

B. What types of lesion do you see in this image? Select MULTIPLE
   1. Active lesion (papules, vesicles, pustule/pseudo-pustule, umbilicated pseudo-pustule, erosion, or ulcer)
   2. Crusted and scabbed lesion (thick surface scale (stratum corneum), yellow serous crust, haemorrhagic crust or scab (eschar)).
   3. Resolved and healed lesion (lesion is no longer raised above the skin and any residual crust has resolved (desquamation)).
   4. Unable to classify

Question 11

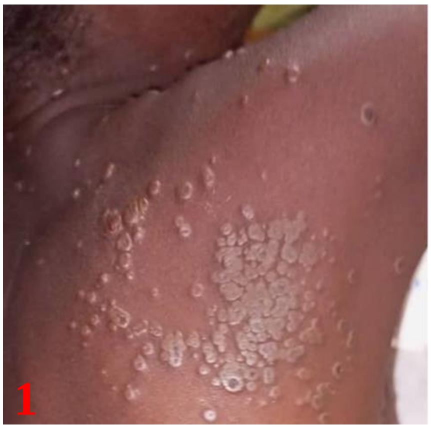

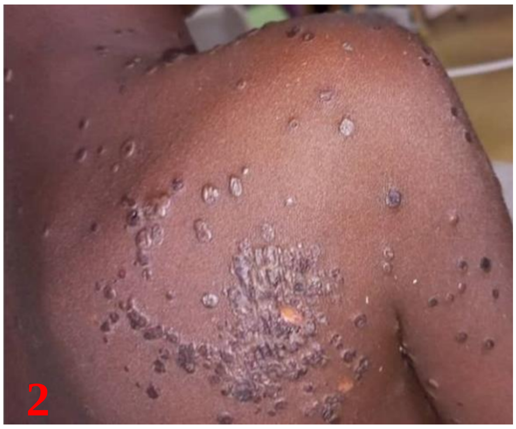

A. Based on the lesion presentation in these images, which of the following diagnoses is more likely? Select ONE
   1. Mpox
   2. Chickenpox (varicella)

B. What types of lesion do you see in image 1? Select MULTIPLE
   1. Active lesion (papules, vesicles, pustule/pseudo-pustule, umbilicated pseudo-pustule, erosion, or ulcer)
   2. Crusted and scabbed lesion (thick surface scale (stratum corneum), yellow serous crust, haemorrhagic crust or scab (eschar)).
   3. Resolved and healed lesion (lesion is no longer raised above the skin and any residual crust has resolved (desquamation)).
   4. Unable to classify

C. What types of lesion do you see in image 2? Select MULTIPLE
   1. Active lesion (papules, vesicles, pustule/pseudo-pustule, umbilicated pseudo-pustule, erosion, or ulcer)
   2. Crusted and scabbed lesion (thick surface scale (stratum corneum), yellow serous crust, haemorrhagic crust or scab (eschar)).
   3. Resolved and healed lesion (lesion is no longer raised above the skin and any residual crust has resolved (desquamation)).
   4. Unable to classify

Question 12

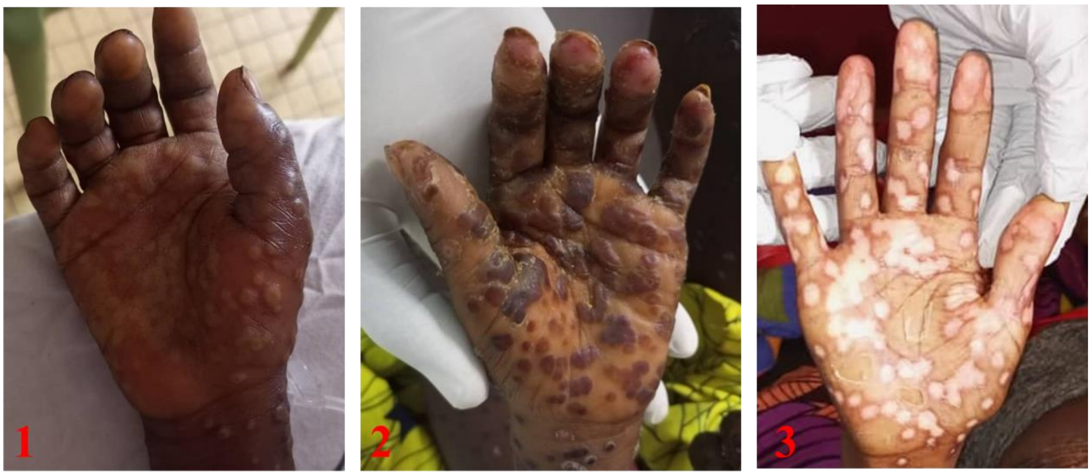

A. Based on the lesion presentation in these images, which of the following diagnoses is more likely? Select ONE
    3. Mpox
    4. Chickenpox (varicella)

B. What types of lesion do you see in image 1? Select ONE
    5. Active lesion (papules, vesicles, pustule/pseudo-pustule, umbilicated pseudo-pustule, erosion, or ulcer)
    6. Crusted and scabbed lesion (thick surface scale (stratum corneum), yellow serous crust, haemorrhagic crust or scab (eschar)).
    7. Resolved and healed lesion (lesion is no longer raised above the skin and any residual crust has resolved (desquamation)).
    8. Unable to classify

C. What types of lesion do you see in image 2? Select ONE
    9. Active lesion (papules, vesicles, pustule/pseudo-pustule, umbilicated pseudo-pustule, erosion, or ulcer)
    10. Crusted and scabbed lesion (thick surface scale (stratum corneum), yellow serous crust, haemorrhagic crust or scab (eschar)).
    11. Resolved and healed lesion (lesion is no longer raised above the skin and any residual crust has resolved (desquamation)).
    12. Unable to classify

D. What types of lesion do you see in image 3? Select ONE
    13. Active lesion (papules, vesicles, pustule/pseudo-pustule, umbilicated pseudo-pustule, erosion, or ulcer)
    14. Crusted and scabbed lesion (thick surface scale (stratum corneum), yellow serous crust, haemorrhagic crust or scab (eschar)).
    15. Resolved and healed lesion (lesion is no longer raised above the skin and any residual crust has resolved (desquamation)).
    16. Unable to classify

Question 13

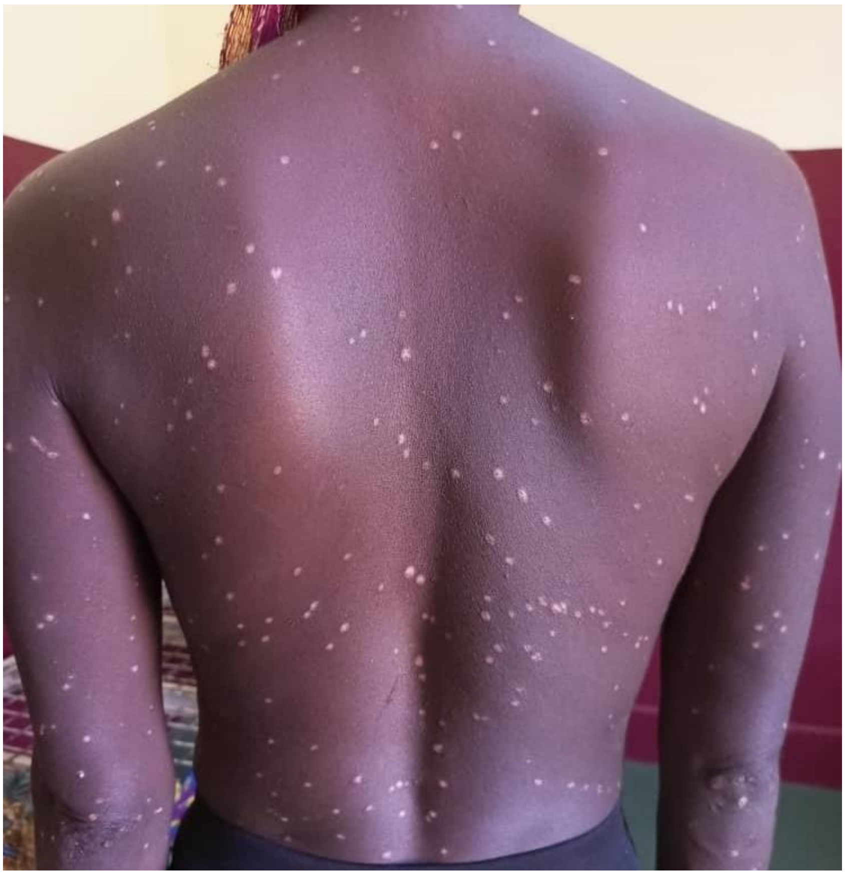

A. Based on the lesion presentation in this image, which of the following diagnoses is more likely? Select ONE
   1. Mpox
   2. Chickenpox (varicella)

B. What types of lesion do you see in this image? Select ONE
   1. Active lesion (papules, vesicles, pustule/pseudo-pustule, umbilicated pseudo-pustule, erosion, or ulcer)
   2. Crusted and scabbed lesion (thick surface scale (stratum corneum), yellow serous crust, haemorrhagic crust or scab (eschar)).
   3. Resolved and healed lesion (lesion is no longer raised above the skin and any residual crust has resolved (desquamation)).
   4. Unable to classify

Question 14

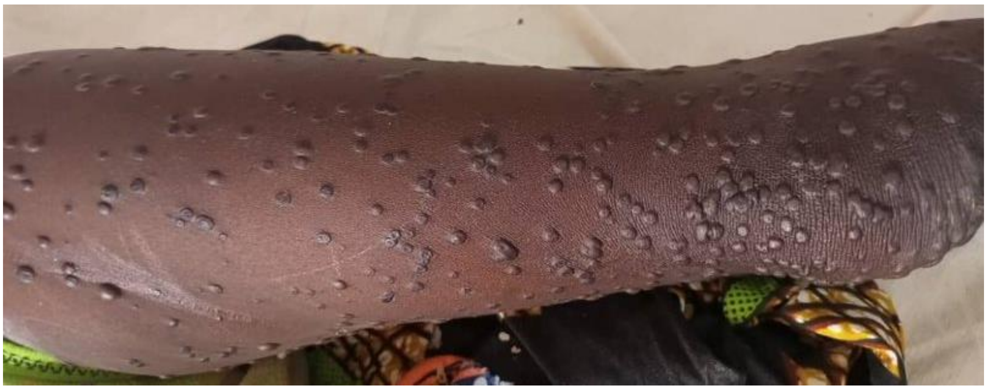

A. Based on the lesion presentation in this image, which of the following diagnoses is more likely? Select ONE
   1. Mpox
   2. Chickenpox (varicella)

B. What types of lesion do you see in this image? Select MULTIPLE
   1. Active lesion (papules, vesicles, pustule/pseudo-pustule, umbilicated pseudo-pustule, erosion, or ulcer)
   2. Crusted and scabbed lesion (thick surface scale (stratum corneum), yellow serous crust, haemorrhagic crust or scab (eschar)).
   3. Resolved and healed lesion (lesion is no longer raised above the skin and any residual crust has resolved (desquamation)).
   4. Unable to classify

Question 15

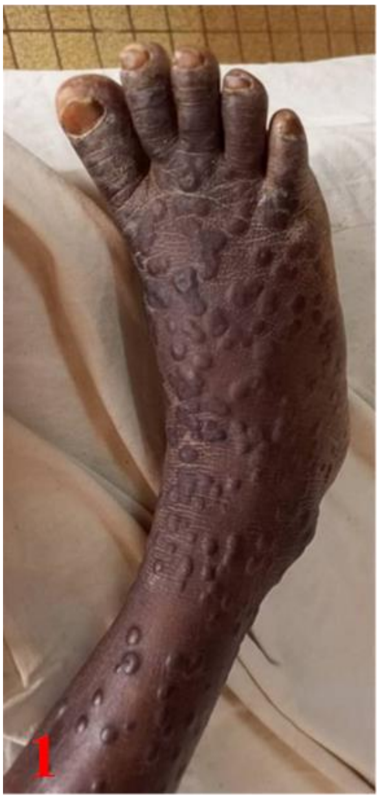

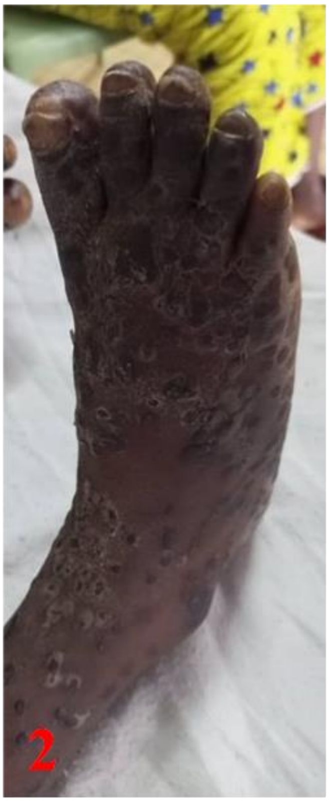

A. Based on the lesion presentation in these images, which of the following diagnoses is more likely? Select ONE
   1. Mpox
   2. Chickenpox (varicella)

B. What types of lesion do you see in image 1? Select ONE
   1. Active lesion (papules, vesicles, pustule/pseudo-pustule, umbilicated pseudo-pustule, erosion, or ulcer)
   2. Crusted and scabbed lesion (thick surface scale (stratum corneum), yellow serous crust, haemorrhagic crust or scab (eschar)).
   3. Resolved and healed lesion (lesion is no longer raised above the skin and any residual crust has resolved (desquamation)).
   4. Unable to classify

C. What types of lesion do you see in image 2? Select ONE
   1. Active lesion (papules, vesicles, pustule/pseudo-pustule, umbilicated pseudo-pustule, erosion, or ulcer)
   2. Crusted and scabbed lesion (thick surface scale (stratum corneum), yellow serous crust, haemorrhagic crust or scab (eschar)).
   3. Resolved and healed lesion (lesion is no longer raised above the skin and any residual crust has resolved (desquamation)).
   4. Unable to classify

Question 16

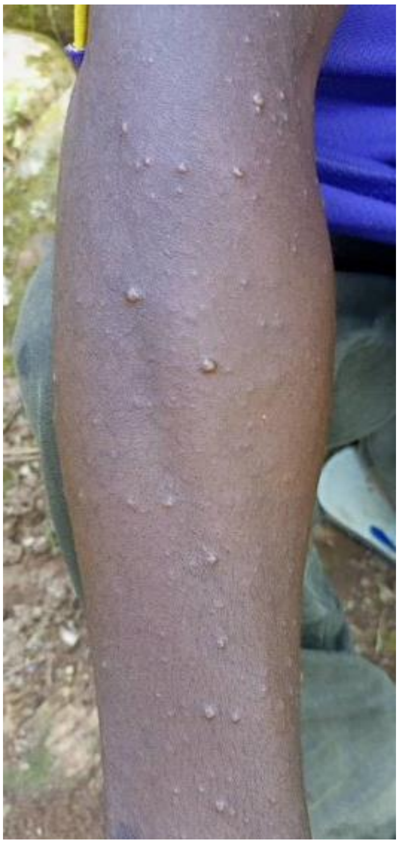

A. Based on the lesion presentation in these images, which of the following diagnoses is more likely? Select ONE
   1. Mpox
   2. Chickenpox (varicella)

B. What types of lesion do you see in this image? Select MULTIPLE
   1. Active lesion (papules, vesicles, pustule/pseudo-pustule, umbilicated pseudo-pustule, erosion, or ulcer)
   2. Crusted and scabbed lesion (thick surface scale (stratum corneum), yellow serous crust, haemorrhagic crust or scab (eschar)).
   3. Resolved and healed lesion (lesion is no longer raised above the skin and any residual crust has resolved (desquamation)).
   4. Unable to classify

Question 17

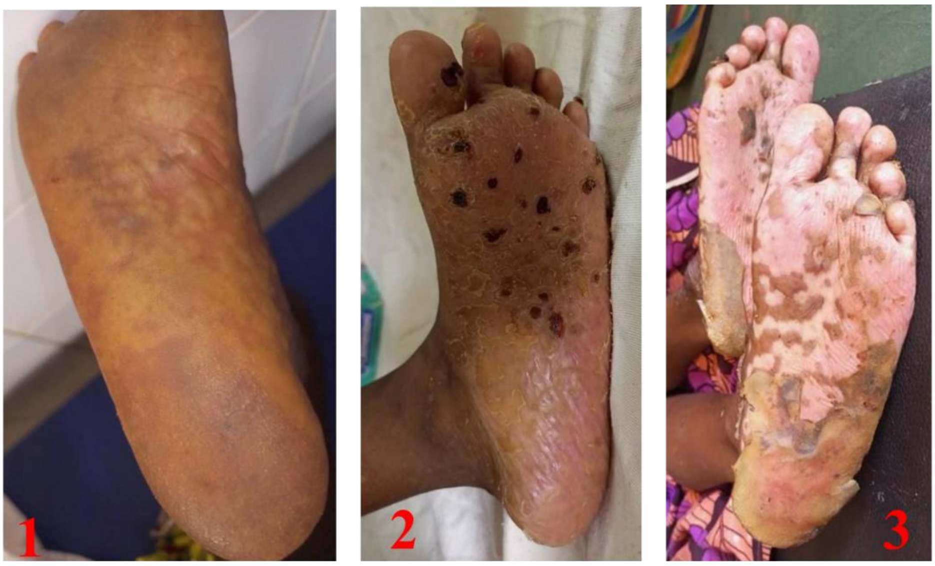

A. Based on the lesion presentation in these images, which of the following diagnoses is more likely? Select ONE
   1. Mpox
   2. Chickenpox (varicella)

B. What types of lesion do you see in image 1? Select ONE
   1. Active lesion (papules, vesicles, pustule/pseudo-pustule, umbilicated pseudo-pustule, erosion, or ulcer)
   2. Crusted and scabbed lesion (thick surface scale (stratum corneum), yellow serous crust, haemorrhagic crust or scab (eschar)).
   3. Resolved and healed lesion (lesion is no longer raised above the skin and any residual crust has resolved (desquamation)).
   4. Unable to classify

C. What types of lesion do you see in image 2? Select ONE
   1. Active lesion (papules, vesicles, pustule/pseudo-pustule, umbilicated pseudo-pustule, erosion, or ulcer)
   2. Crusted and scabbed lesion (thick surface scale (stratum corneum), yellow serous crust, haemorrhagic crust or scab (eschar)).
   3. Resolved and healed lesion (lesion is no longer raised above the skin and any residual crust has resolved (desquamation)).
   4. Unable to classify

E. What types of lesion do you see in image 3? Select ONE
   1. Active lesion (papules, vesicles, pustule/pseudo-pustule, umbilicated pseudo-pustule, erosion, or ulcer)
   2. Crusted and scabbed lesion (thick surface scale (stratum corneum), yellow serous crust, haemorrhagic crust or scab (eschar)).
   3. Resolved and healed lesion (lesion is no longer raised above the skin and any residual crust has resolved (desquamation)).
   4. Unable to classify

Question 18

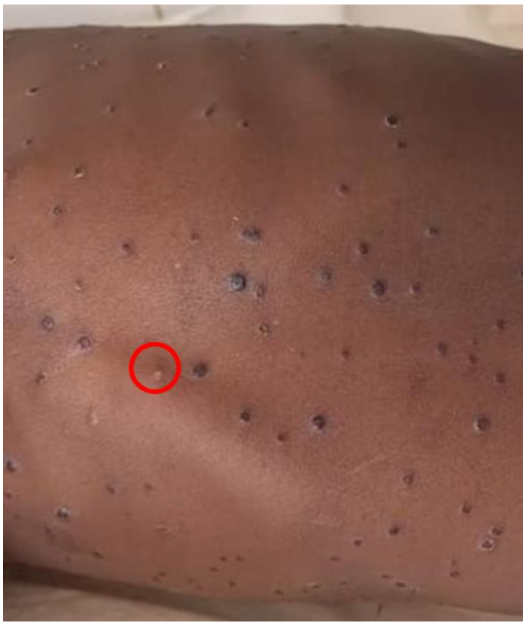

A. Based on the lesion presentation in this image, which of the following diagnoses is more likely? Select ONE
   1. Mpox
   2. Chickenpox (varicella)

B. What type of lesion do you see circled in red in this image? Select ONE
   1. Active lesion (papules, vesicles, pustule/pseudo-pustule, umbilicated pseudo-pustule, erosion, or ulcer)
   2. Crusted and scabbed lesion (thick surface scale (stratum corneum), yellow serous crust, haemorrhagic crust or scab (eschar)).
   3. Resolved and healed lesion (lesion is no longer raised above the skin and any residual crust has resolved (desquamation)).
   4. Unable to classify

Question 19

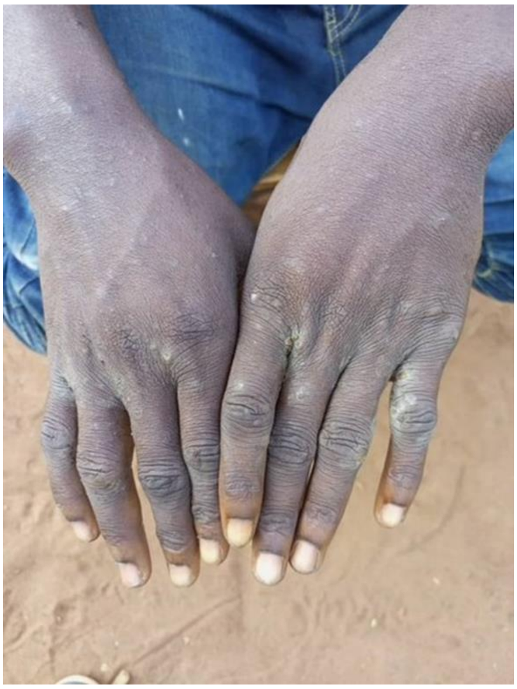

A. Based on the lesion presentation in this image, which of the following diagnoses is more likely? Select ONE
   1. Mpox
   2. Chickenpox (varicella)

B. What types of lesion do you see in this image? Select MULTIPLE
   1. Active lesion (papules, vesicles, pustule/pseudo-pustule, umbilicated pseudo-pustule, erosion, or ulcer)
   2. Crusted and scabbed lesion (thick surface scale (stratum corneum), yellow serous crust, haemorrhagic crust or scab (eschar)).
   3. Resolved and healed lesion (lesion is no longer raised above the skin and any residual crust has resolved (desquamation)).
   4. Unable to classify

Question 20

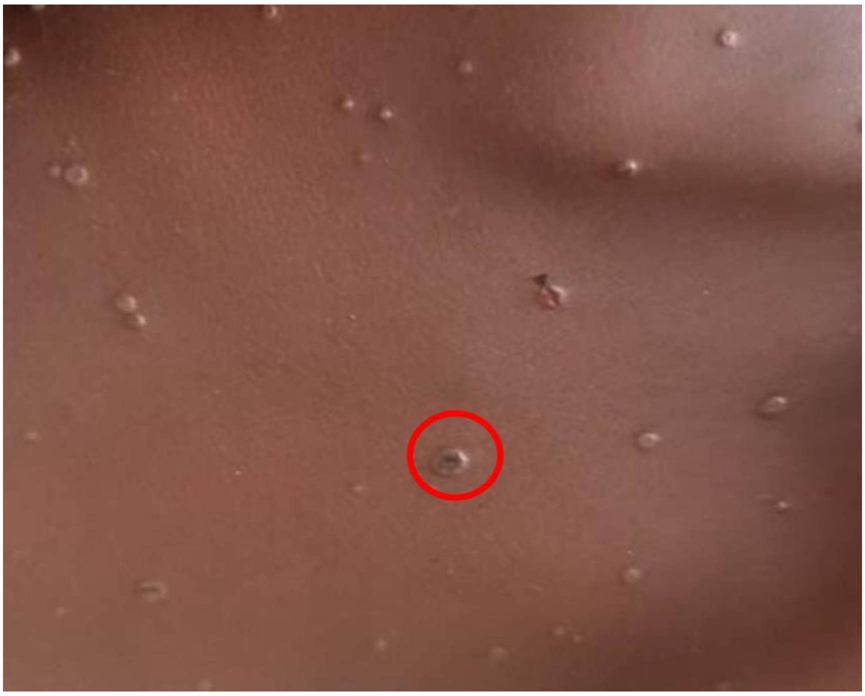

A. Based on the lesion presentation in this image, which of the following diagnoses is more likely? Select ONE
   1. Mpox
   2. Chickenpox (varicella)

B. What type of lesion do you see circled in red in this image? Select ONE
   1. Active lesion (papules, vesicles, pustule/pseudo-pustule, umbilicated pseudo-pustule, erosion, or ulcer)
   2. Crusted and scabbed lesion (thick surface scale (stratum corneum), yellow serous crust, haemorrhagic crust or scab (eschar)).
   3. Resolved and healed lesion (lesion is no longer raised above the skin and any residual crust has resolved (desquamation)).
   4. Unable to classify
