# Supplementary material for: Challenges in clinical diagnosis of Clade I Mpox: Highlighting the need for enhanced diagnostic approaches
Source: PLoS Negl Trop Dis. 2024 Jun 24;18(6):e0012087. doi: 10.1371/journal.pntd.0012087 (PMC11226010; doi:10.1371/journal.pntd.0012087)
Supplement: S2 Appendix — (PDF) [file pntd.0012087.s002.pdf]

Appendix 2 – Summary of question characteristics and accuracy score

Question	Accuracy (%)	Diagnosis	Lesion type(s)
Question 4	25	Mpox	Multiple
Question 19	43.75	Mpox	Multiple
Question 5	50	Varicella	Single
Question 16	56.25	Varicella	Multiple
Question 11	62.5	Mpox	Multiple
Question 9	68.75	Varicella	Multiple
Question 13	68.75	Mpox	Single
Question 18	68.75	Mpox	Single
Question 20	75	Mpox	Single
Question 6	81.25	Mpox	Single
Question 8	81.25	Mpox	Multiple
Question 10	81.25	Mpox	Multiple
Question 14	93.75	Mpox	Multiple
Question 17	93.75	Mpox	Single
Question 7	100	Mpox	Single
Question 12	100	Mpox	Single
Question 15	100	Mpox	Single
