# Supplementary material for: Challenges in clinical diagnosis of Clade I Mpox: Highlighting the need for enhanced diagnostic approaches
Source: PLoS Negl Trop Dis. 2024 Jun 24;18(6):e0012087. doi: 10.1371/journal.pntd.0012087 (PMC11226010; doi:10.1371/journal.pntd.0012087)
Supplement: Attachment — Submitted filename: Response to reviewers_15May2024.docx [file pntd.0012087.s004.docx]

15 May 2024

Dear Reviewers,

**Re: Challenges in Clinical Diagnosis of Clade I Mpox: Highlighting the Need for Enhanced Diagnostic Approaches**

Thank you very much for providing helpful feedback on our article. Please find below our response to each of your comments and a summary of the changes made to the manuscript.

| **Reviewer comment** | **Author response** |
| --- | --- |
| The desired sample size was not defined. | We have now included in the manuscript a) we did not define a sample size before the study was conducted and b) the reason why we did not define a target sample size (that there is a limited pool of clinicians with experience of clade I mpox assessment). |
| Several of the figures seem of limited value to me, without adding to the information in the narrative and tables, if the number of figures is an issue for the publisher. | We have improved the figures associated with this manuscript to be more informative and easier to read |
| There needs to be more detail about the makeup of the persons who were sent the survey, not just a table of where respondents were based and self-report on expertise and number of patients treated. | We have provided more details about the country of work of the clinicians contacted to participate and added a summary figure. |
| Reviewer #1: There should be an acknowledgement that clinicians have more data than just the appearance of the lesions when making a diagnosis. The WHO definition is not only based on the appearance of the lesions, as the authors suggest, but also on information that help judge the likelihood of alternate diagnoses.  Reviewer #2: However, it's important to acknowledge that diagnosis is multifaceted, relying not only on images but also on factors like risk assessment, patient history, and physical examination. While the study emphasizes the role of images, it should discuss the broader diagnostic process in its introduction and discussion. | We agree and hope this is more evident in the expanded text in the revision. In particular we now use the more specific term ‘visual’ assessment rather than ‘clinical’ assessment, that, as you point out, brings in many other facets.  From line 24 – 26 we now include the text ‘While clinical diagnosis is multifaceted and takes into consideration factors such as patient demographics, prevailing epidemiology, and clinical history – one important component is visual assessment of skin lesions. ‘  On lines 187 and 188 we have expanded the list of other factors taken into consideration when making a clinical diagnosis (in the absence of point of care diagnostic tests). |
| Additionally, exploring prior research on the role of images in diagnosing diseases like mumps, smallpox, and varicella, which primarily present with characteristic rashes, would enhance the study's context and significance. | We agree with this. Interestingly, we weren’t able to find analogous studies in the literature assessing these lesions. We suggest this might be due to better natural history data for these diseases (e.g. presence of other diagnostic discriminators such as parotitis, or rash pattern, or epidemiology), and, in high-income settings, the availability of diagnostic tests. We’ve added text to the discussion (lines 172-176) with this point. |

Sincerely,

Josephine Bourner
